# Benign multiple sclerosis and long-term outcomes after 30 years

**DOI:** 10.1007/s00415-025-13402-8

**Published:** 2025-11-05

**Authors:** E. Matas, L. Bau, L. Romero-Pinel, I. León, P. Arroyo-Pereiro, A. Muñoz-Vendrell, A. Martínez-Yélamos, S. Martínez-Yélamos

**Affiliations:** 1 Multiple Sclerosis Unit, Department of Neurology, Hospital Universitari de Bellvitge, L’Hospitalet de Llobregat, Feixa Llarga s/n, 08907 Barcelona, Spain; 2 Neurologic Diseases and Neurogenetics Group, Neuroscience Program, Institut d’Investigació Biomèdica de Bellvitge (IDIBELL), L’Hospitalet de Llobregat, Barcelona, Spain; 3 Departament de Ciències Clíniques, Facultat de Medicina, Universitat de Barcelona, Barcelona, Spain

**Keywords:** Benign multiple sclerosis, Cognition, Prognosis, Long-term outcome

## Abstract

**Background:**

The concept of benign multiple sclerosis (MS) is a matter of debate. In most definitions, only the Expanded Disability Status Scale (EDSS) score and disease duration are considered, although other factors may influence a patient’s disability. The aims of this study were to evaluate the percentage of patients with benign MS in our cohort and to determine the proportion of these patients who remained benign after 20 and 30 years of disease evolution. Clinical and demographic variables related to this outcome were investigated.

**Methods:**

Patients fulfilling the criteria for benign MS (EDSS < 3.0 and at least 10 years of MS evolution) were selected from a hospital-based series and followed up prospectively. The EDSS was assessed after 20 and 30 years of MS evolution. Clinical and radiological variables were evaluated. Fatigue, depression and cognition tests were performed.

**Results:**

Eighty-two of the 485 patients evaluated in 1996 fulfilled the criteria for benign MS and were selected. In 51 of the 68 (75%) patients evaluated in 2006, the EDSS score continued to be ≤ 3. In 2016, 35 out of 58 (60%) patients continued to have an EDSS score ≤ 3. None of the clinical variables could predict persistence in the benign group. Eighteen of the 35 benign MS patients completed the questionnaires after 30 years. Fifty percent of them reported fatigue, 22% depression, and 83% had cognitive impairment.

**Conclusion:**

The majority of MS patients maintained a benign status even after 30 years of disease evolution. However, cognitive impairment in this group is notable.

## Introduction

Multiple sclerosis (MS) is a chronic demyelinating disease of the central nervous system with a wide range of manifestations. The progression of disability in MS is highly variable, ranging from a mild course to a severe and disabling condition. The concept of benign MS is a matter of controversy. Several definitions of a benign MS course have been used in recent years, the most common being a score of ≤ 3 on the Expanded Disability Status Scale (EDSS) [1] 10 years after disease onset [2].

Patients with an apparently benign MS course may experience a subsequent disability. A frequent question posed by clinicians is whether benign MS is stable or a condition that can change to disabling disease as the follow-up time increases.

The reported incidence of benign MS ranges from 6 to 64%, depending on the definition used and the follow-up time [3–5].

Moreover, the EDSS score does not adequately evaluate symptoms such as fatigue or cognitive impairment, which can affect MS patients’ quality of life. Owing to this undervaluation, patients with benign MS can experience considerable fatigue.

Nonetheless, in the treatment era, the concept of benign MS may be uncertain. It’s important to consider the extent to which the therapeutic benefit of early disease-modifying treatment in patients with MS is due to the inherent benignity of the disease or to the benefit of the treatment.

The aims of this study were to evaluate the percentage of patients with benign MS in our cohort and to determine the proportion of these patients who remained benign after 20 and 30 years of disease evolution. Clinical and demographic variables related to this outcome were investigated.

## Patients and methods

### Study population

Patients were selected from a hospital-based series of MS patients who attended the MS clinic of Bellvitge University Hospital. Our MS clinic is the reference centre for demyelinating diseases in the health district of Gerencia Territorial Barcelona Metropolitana Sud in Catalonia, a region in northeast Spain. Patients were registered using the European Database for Multiple Sclerosis (EDMUS) [6] and followed up on a 6-month basis and at the time of relapse. Demographic and clinical data, including disability, were recorded at each visit. The study population included all patients registered in our EDMUS database up to 1 July 1996, corresponding to 485 patients.

From the total population, patients who met the following criteria were selected: clinically definite MS according to the Poser criteria [7], relapsing remitting form, and fulfilling the criterion for benign MS: an EDSS score ≤ 3 after at least 10 years of disease evolution. The EDSS score had to be confirmed at least 6 months later to be defined as irreversible [8]. These patients were considered to have a benign MS course at 10 years. In order to facilitate comparision with other studies that use different EDSS scores to define benign MS, patients were classified into 4 subgroups according to their EDSS score at 10 years after onset: EDSS score ≤ 3, EDSS score ≤ 2, EDSS score ≤ 1 and EDSS score = 0.

Data from patients in the benign MS course group at 10 years were assessed again 10 years and 20 years later. Evaluations prospectively included in the EDMUS database were used for this purpose. Patients were considered to have benign MS at 20 years if their residual EDSS score continued to be ≤ 3 at least 20 years after MS onset and benign MS at 30 years if this condition was maintained 30 years after MS onset.

In addition, a comparative analysis of clinical and radiological variables was conducted between benign MS patients after 20 and 30 years and those who had cesated to be benign in each period.

Patients with benign MS at 30 years were asked to evaluate their cognition, depression and fatigue. A group of MS patients matched by sex and age, with an EDSS score ≤ 3 and with less than 20 years of MS evolution were selected as the control group.

The study was approved by the Hospital Universitari de Bellvitge Research Ethics Committee.

### Variables

The following variables were analysed: age at onset; sex; symptoms at onset (monoregional or polyregional onset and functional system affected according to Kurtzke); EDSS score in 1996, 2006 and 2016; time to increase 1 point on the EDSS score from 1996; number of relapses in the first 2 years of disease; time to second relapse; use of disease-modifying drugs; presence of oligoclonal bands or a high IgG index; visual evoked potentials; and fulfilment of the Barkhof criteria on magnetic resonance imaging (MRI).

### Tests

Tests to evaluate fatigue, depression and cognition were performed for benign MS patients after at least 30 years of MS evolution. Tests to evaluate cognition were also performed for MS controls.

The Modified Fatigue Impact Scale (MFIS) is a patient-reported measure used to evaluate fatigue. It contains 21 items scored from 0 to 4 points. Higher scores reflect greater fatigue, and a total score of 38 is considered the cut-off indicating relevant fatigue [9].

The Patient Health Questionnaire (PHQ-9) is a 9-item self-reported test used to evaluate depression. Each of the 9 items is scored from 0 to 3 points. Higher scores indicate greater symptoms of depression. A score equal to or greater than 10 is considered to indicate significant depression [10]

The Symbol Digit Modalities Test (SDMT) is an oral or written test widely used to evaluate cognitive processing efficiency and speed in MS patients. It can be used as a screening tool for cognitive impairment [11]. Oral versions of the test were administered to patients and controls. The final score is the correct number of items answered in a period of 90 s [12]. A score of 49 or lower was considered the best for identifying cognitive impairment in our population [13].

### Statistical analysis

Variables were tested for normality. The chi-square or Fisher exact’s test and Student’s t test or Mann-Whitney *U* test were used to compare categorical and continuous variables, respectively. A p value of < 0.05 was considered to indicate statistical significance for each comparison. In the analysis of prognostic variables, Bonferroni correction was applied to adjust for multiple comparisons. In this instance, the p-value must be < 0.003 to achieve statistically significance.

## Results

**Fig. 1:**
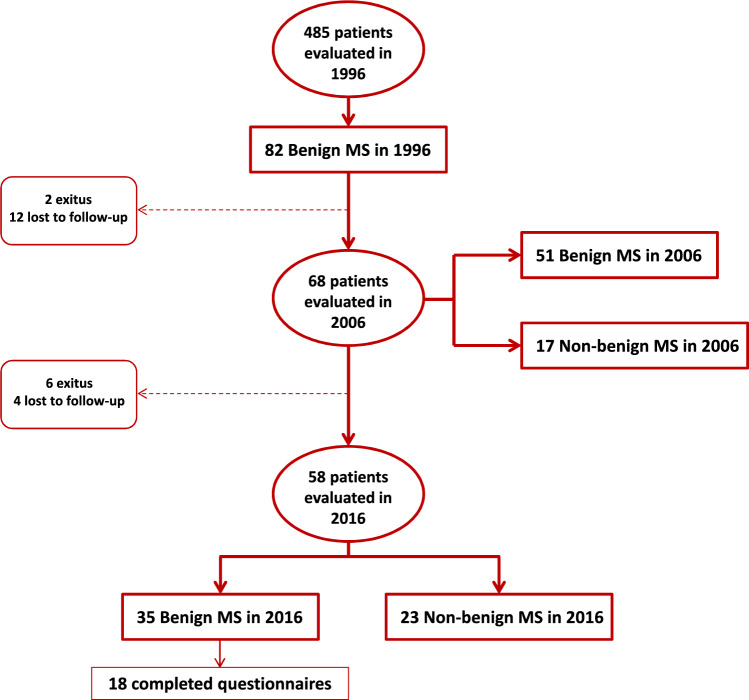
Flow chart showing patient follow-up for up to 30 years

Eighty-two of the 485 patients registered in our hospital database in 1996 fulfilled the inclusion criteria (Fig. 1). Analysis of the demographic characteristics of these 82 patients revealed that the majority were women (63%) and the mean age was 42 years. The median disease duration was 15.4 years, and the mean age at onset was 25 years. The median EDSS score in 1996 was 1,0, and the mean number of relapses from onset was 6.2 (Table 1).

**Table 1:** Baseline característics of benign MS patients in 1996

	Benign MS in 1996
Number of patients	82
Sex (female)	63%
Age, mean (SD)	42.7 (9.6)
Time MS evolution, median (IQR)	14.7 (8.3)
Age at onset, mean (SD)	24.9 (9.6)
Relapses from onset, mean (SD)	6.2 (4.5)
EDSS in 1996, median (IQR)	1,0 (0–3)

Among these 82 patients, 68 were still being followed up in our centre in 2006, and 14 were lost to follow-up: 2 patients changed their city of residence, one died due to lymphatic leukaemia, one died due to bronchial aspiration, and 10 patients withdrew from follow-up for unknown reasons. At the last medical examination of these 14 patients, the median EDSS score was 3.0.

In 51 of the 68 (75%) patients evaluated after more than 20 years of disease evolution, the EDSS score continued to be ≤ 3. In the worst-case scenario, in which patients lost to follow-up were considered to have non-benign MS, the percentage of patients with an EDSS score ≤ 3 was 62.1% (51 of 82).

In 2016, 58 of the 82 patients were evaluated. Ten patients were lost to follow-up since 2006: two patients changed their residence, six patients died (stroke, meningitis, lung cancer, mandibular cancer, breast cancer and renal cancer), and two patients were lost to follow-up for unknown reasons. At the last medical examination of these 10 patients, the median EDSS score was 6.0.

After at least 30 years of MS evolution, 35 of the 58 patients evaluated continued to have an EDSS of three or less (60%). In the worst-case scenario (patients lost to follow-up considered to have non-benign MS), the percentage of patients with an EDSS score ≤ 3 was 42.7% (35 of 82).

Subgroups according to EDSS scores after 10 years of MS evolution were similarly evaluated, and the results are shown in Table 2. Among the subgroup of patients whose EDSS score was ≤ 2 after 10 years of MS evolution, the percentage of patients remaining in the benign group was 83.7% (41 of 49) after 20 years and 65.9% (29 of 44) after 30 years.

**Table 2:** Assessment of patients with benign multiple sclerosis after 20 and 30 years of follow-up

EDDS score in 1996	Number of patients in 1996	Patients lost from 1996 to 2006	Number of patients (%) followed-up in 2006	Benign MS patients (EDSS ≤ 3) (%) in 2006	Patients lost from 1996 to 2016	Number of patients (%) followed-up in 2016	Benign MS patients (EDSS ≤ 3) (%) in 2016
EDSS ≤ 3.0	82	14	68 (82.9%)	51 (75.0%)	24	58 (70.7%)	35 (60.0%)
EDSS ≤ 2.0	58	9	49 (84.5%)	41 (83.7%	14	44 (75.9%)	29 (65.9%)
EDSS ≤ 1.0	42	7	35 (83.3%)	31 (88.6%)	9	33 (78.6%)	23 (69.7%)
EDSS = 0	34	7	27 (79.4%)	23 (85.2%)	9	25 (73.5%)	15 (60.0%)

### Prognostic variables

Data from 68 patients followed in 2006 were obtained from the EDMUS database (Table 3).

**Table 3:** Prognostic variables in the benign and non-benign multiple sclerosis groups after 20 years of disease evolution

Variable	Benign MS in 2006 EDSS ≤ 3	Non-benign MS in 2006 EDSS > 3	*P* value^a^	*P* value^b^
Number of patients	51	17	–	–
Mean age at onset (SD)	26.44 (8.64)	18.87 (7.13)	0.002^+^	< 0.003
Sex				
Men	18 (35.3%)	6 (35.3%)	1.0^*^	ns
Women	33 (64.7%)	11 (64.7%)		
EDSS in 1996				
0	23 (45.1%)	4 (23.5%)		
1	8 (15.7%)	0 (0.0%)	0.025^*^	ns
2	10 (19.6%)	4 (23.5%)		
3	10 (19.6%)	9 (52.9%)		
Onset				
Monoregional	35 (68.6%)	16 (94.1%)	0.051^*^	ns
Polyregional	16 (31.4%)	1 (5.9%)		
Immunomodulatory drugs				
No	29 (56.9%)	3 (17.6%)	0.006^*^	ns
Yes	22 (43.1%)	14 (82.4%)		
Oligoclonal bands/IgG index				
Negative Positive	11 (28.2%) 28 (71.8%)	5 (33.3%) 10 (66.7%)	0.71^*^	ns
Visual evoked potentials				
Normal	5 (10.6%)	1 (5.9%)	1.0^*^	ns
Positive	42 (89.4%)	16 (94.1%)		
MRI: Barkhof criteria at onset				
1st positive (≥ 9 T2 lesions)	42 (91.3%)	17 (100%)	0.57^*^	ns
2nd positive (≥ 1 infratentorial)	33 (64.7%)	13 (76.5%)	0.55	ns
3rd positive (≥ 1 juxtacortical)	37 (86%)	9 (52.9%)	1.0	ns
4th positive (≥ 3 periventricular)	47 (95.9%)	17 (100%)	1.0	ns
Relapses during the first 2 years, mean (SD)	1.47 (1.06)	1.12 (0.33)	0.18^+^	ns
Time between onset and next relapse (years), mean (SD)	6.99 (5.49)	8.66 (7.58)	0.33^+^	ns
Time between onset and year 2006 (years), mean (SD)	26.54 (5.22)	30.9 (7.28)	0.03^+^	ns
Time to increase 1 point on the EDSS score (years), mean (SD)	4.26 (2.75)	3.76 (2.93)	0.62^+^	ns
Functional systems affected at onset				
Optic neuritis	12 (23.5%)	2 (11.8%)	0.49^*^	ns
Sensory	19 (37.3%)	5 (29.4%)	0.56^*^	ns

^*^ Chi-square test or Fisher’s exact test, as appropriate

^+^ Student’s *t*-test or Mann-Whitney *U* test, as appropriate

*Ns* not significant

^a^ Before Bonferroni correction

^b^ After Bonferroni correction

The mean age at onset was 26.4 years in the 51 patients who maintained an EDSS score ≤ 3 at 20 years (benign group) and 18.9 years in the 17 patients with an EDSS score > 3 at 20 years (non-benign group), with significant differences remaining after Bonferroni adjustment (p = 0.002). The time of MS evolution (time between onset and 2006) was longer in non-benign MS patients than in benign MS patients (median (interquartile range (IQR)) 31.8 (12.6) vs 25.8 (7.9) years). The difference reached statistical significance but did not persist after Bonferroni adjustment (p = 0.03).

There were no significant differences between the EDSS score (0, 1, or 2) in 1996 and the probability of having benign or non-benign MS in 2006. Monoregional or polyregional onset of disease was not related to the probability of maintaining an EDSS score ≤ 3 at 20 years. The mean number of relapses during the first 2 years was similar in both groups.

In relation to the functional system affected, we evaluated the onset of MS with optic neuritis and sensory symptoms, as these manifestations are often related to a benign course. There were no differences between the two groups in relation to these symptoms at onset.

Thirty-six (53%) of the patients followed in 2006 had been treated with immunomodulatory drugs (35 patients had received Interferon at some point, 6 Glatiramer Acetate, 4 Natalizumab, 2 Fingolimod and 2 Mitoxantrone). None of the patients had been treated with these drugs in our hospital before 1996. In total, 43.1% of the benign patients and 82.4% of the non-benign patients had received disease-modifying treatment at any time. The differences did not reach statistical significance after Bonferroni adjustment (p = 0.006).

Data on the detection of oligoclonal bands and the IgG index were available for 79.4% of patients, and visual evoked potentials were available for 94.1%. Analysis of these data revealed no significant differences between the groups.

The first MRI studies were evaluated for all patients, except for nine patients for whom MRI scans were not available. Findings consistent with the Barkhof criteria were investigated in each MRI scan. Limitations due to the quality of the scans (some of them from the 1980 s) must be considered. The evaluation revealed no differences between the benign and non-benign groups.

A similar analysis was performed for patients after at least 30 years of disease evolution. Data from 58 patients followed in 2016 were also obtained from the EDMUS database (Table 4).

**Table 4:** Prognostic variables in the benign and non-benign multiple sclerosis groups after 30 years of disease evolution

Variable	Benign MS in 2016 EDSS ≤ 3	Non-benign MS in 2016 EDSS > 3	*P* value^a^	*P* value^b^
Number of patients	35	23	–	–
Mean age at onset (SD)	26.94 (8.84)	21.08 (7.56)	0.01^+^	ns
Sex				
Men	12 (34.3%)	9 (39.1%)	0.71^*^	ns
Women	23 (65.7%)	14 (60.8%)		
EDSS in 1996				
0	15 (42.9%)	10 (43.5%)		
1	8 (22.9%)	0 (0.0%)	0.066^*^	ns
2	6 (17.1%)	5 (21.7%)		
3	6 (17.1%)	8 (34.8%)		
Onset				
Monoregional	25 (71.4%)	18 (78.3%)	0.56^*^	ns
Pluriregional	10 (28.6%)	5 (21.7%)		
Immunomodulatory drugs				
No	22 (62.9%)	5 (21.7%)	0.002^*^	< 0.003
Yes	13 (37.1%)	18 (78.3%)		
Oligoclonal bands/IgG index				
Negative	9 (32.1%)	4 (21.1%)	0.515^*^	ns
Positive	19 (67.9%)	15 (78.9%)		
Visual evoked potentials				
Normal	2 (6.1%)	4 (19.1%)	0.19^*^	ns
Positive	31 (93.9%)	17 (80.9%)		
MRI: Barkhof criteria at onset				
1st positive (≥ 9 T2 lesions)	30 (90.9%)	20 (100%)	0.28^*^	ns
2nd positive (≥ 1 infratentorial)	23 (65.7%)	17 (73.9%)	0.51	ns
3rd positive (≥ 1 juxtacortical)	27 (90%)	13 (81.2%)	0.40	ns
4th positive (≥ 3 periventricular)	33 (97.1%)	22 (100%)	1.0	ns
Relapses during the first 2 years, mean (SD)	1.57 (1.24)	1.17 (0.39)	0.14^+^	ns
Time between onset and next relapse (years), mean (SD)	7.18 (5.76)	7.0 (4.54)	0.91^+^	ns
Time between onset and year 2016 (years), mean (SD)	37.45 (5.18)	39.38 (6.48)	0.26^+^	ns
Time to increase 1 point on the EDSS score (years), mean (SD)	5.95 (3.57)	5.53 (4.63)	0.8^+^	ns
Functional systems affected at onset				
Optic neuritis	8 (22.9%)	4 (17.4%)	0.75^*^	ns
Sensory	11 (31.4%)	7 (30.4%)	0.94^*^	ns

^*^ Chi-square test or Fisher’s exact test, as appropriate

^+^ Student’s *t*-test or Mann-Whitney* U* test, as appropriate

*Ns* not significant

^a^ Before Bonferroni’s correction

^b^ After Bonferroni’s correction

Notably, the mean age at onset was 26.9 years in 35 benign MS patients and 21.1 years in 23 non-benign MS patients in 2016. The results did not reach statistical significance after Bonferroni adjustment (p = 0.01), unlike the same comparison performed in 2006. The time of MS evolution (time between onset and 2016) was longer in the group of non-benign MS patients than in the group of benign MS patients (median (IQR): 39.0 (11.0) vs 37.0 (7.3) years); however, the difference did not reach statistical significance (p = 0.26).

With respect to treatments, 31 of 58 (53.4%) patients evaluated in 2016 had received MS treatment at any time (30 had been treated with Interferon at some point, 6 Glatiramer Acetate, 4 Natalizumab, 2 Fingolimod and 2 Mitoxantrone). As expected, more non-benign MS patients (78.3%) than benign MS patients (37.1%) had been treated. The result was significant after Bonferroni adjustment (p = 0.002).

No other significant differences were found in the variables analysed in patients after 20 years and after 30 years of MS evolution.

### Questionnaire results

Eighteen patients agreed to undergo the tests to evaluate depression, fatigue and cognition.

Nine out of 18 (50%) patients with benign MS after at least 30 years of MS evolution achieved a score of 38 or higher on the MFIS test and were therefore considered to have fatigue.

When depression was evaluated through the PHQ-9 test, 4 out of 18 (22%) benign MS patients obtained a score of 10 or higher and were subsequently considered to have depression.

The mean SDMT score in the benign MS group was 34.28 (standard deviation (SD) 3.25). Fifteen out of 18 patients (83%) scored 49 or less on the SDMT and were therefore considered to have cognitive impairment.

The SDMT was also administered to the control MS group. Eighteen patients were matched by age and sex. All patients had an EDSS score ≤ 3.0. The median EDSS score was 2.25 ( IQR 1.5–2.62) at that time. The mean duration of MS evolution when the test was performed was 12.06 (SD 5.87) years. The mean SDMT score in the control group was 47.0 (SD 2.28). Ten out of 18 controls (55.6%) scored 49 or less on the SDMT.

The mean SDMT score in the control group was greater than that in the benign MS group (47.0 vs. 34.28). The difference was statistically significant (p = 0.009, Mann‒Whitney *U* test). No significant differences were found in the percentage of patients with cognitive impairment (defined as an SDMT score ≤ 49) between the benign MS group and the control group (p = 0.15, Fisher’s exact test).

## Discussion

The incidence of benign MS varies considerably [5, 14]. This difference could be explained by different factors, such as the diagnostic MS criteria used (Poser vs. McDonald), the type of study (epidemiological vs. hospital-based), the benign MS definition used and the different times at which the study was performed. In our cohort, the group with benign MS (defined on the basis of an EDSS score ≤ 3 after more than 10 years of disease evolution) represented 16.9% of patients followed-up in 1996. Studies using the same definition have variable percentages, ranging from 12.5 to 38.1% [2, 15–20].

MS patients from a hospital-based series were selected for our study. The information on this cohort came from the EDMUS database, in which the data had been prospectively entered in a systematic and standardized manner since the patient’s first visit. As the information was obtained from a hospital-based series, there may have been some selection bias towards more severe cases. Despite this possible bias, our analyses revealed a high probability of remaining in a benign MS course (EDSS score ≤ 3) after more than 20 and 30 years of disease evolution if the EDSS score at 10 years was ≤ 3.

According to our findings, the MS of nearly three-quarters of patients with an EDSS score ≤ 3 after 10 years will remain benign after 20 years of follow-up. Our series showed figures similar to those of other studies using the same benign MS definition, with probabilities ranging from 52 to 69% [15, 16, 18, 21]. Other studies revealed lower probabilities (28%) [2], possibly related to the lower number of patients evaluated and because the studies were conducted at different times.

When an EDSS score ≤ 2 after 10 years is considered, the percentage is even more favourable than the figure achieved by the EDSS of 3. The results are comparable to those of other series, with results ranging between 68 and 93% [15, 18, 21, 22]. Hence, it seems that a lower EDSS score leads to a higher probability of the benign form persisting.

When a longer follow-up period was considered, the percentage of patients in whom a benign course persists decreased. In our study, moret han a half of patients with benign MS in 1996 continued to have a benign course in 2016. Few studies have involved such long follow-up times. Almost all showed lower percentages, approximately 11.4% [23] or 15% [17].

There are no clear prognostic factors for predicting benign MS at the beginning of the disease course [7, 16, 23, 24]. Some studies suggest that female sex, younger age at onset, full recovery after the first relapse, a high frequency of relapses during the first years of disease and onset with optic neuritis or sensory symptoms are associated with a benign MS course [2, 3, 16, 24–29]. Other studies did not reveal such associations [18, 29, 30]. This discrepancy suggests that clinical predictors are not consistent and are difficult to apply individually.

In our study, an evaluation of clinical characteristics and different variables at onset was performed. Patients with a benign course after 20 years of MS evolution were older at onset than patients with a non-benign course were. This finding could be explained by the fact that non-benign patients in 2006 had a longer MS duration and therefore a greater probability of relapse and a higher EDSS score. Although the difference in the time of MS evolution did not reach statistical significance, this explanation for this difference seems reasonable. No other variables showed statistical significance in the 2006 evaluation.

In the analysis of prognostic factors in 2016, the variable that reached statistical significance after Bonferroni correction was immunomodulatory treatment. A total of 78% of non-benign patients received any MS treatment, whereas 37% of benign MS patients did. This difference seems to be a result and not a cause of higher EDSS scores: patients with severe disease are more likely to be treated and, nevertheless, to have worsened disability. Hence, we have not been able to find any clear variable that could predict which patients will continue to have a benign course after 10 or 20 years.

These results should be considered before therapeutic decisions are made, especially in the application of new emerging therapies. In general, the more effective these treatments are, the greater their potential for side effects. For this reason, determining the risk of disability progression has become an important issue when choosing the best treatment option. Predicting which patients are going to have a benign form of MS would be desirable to help in the decision of whether to treat from the beginning of the disease. However, the diagnosis of benign MS can be made only after more than 10 years of disease evolution, and even after this period, it may be too early to predict disease evolution, as the patient’s status may be temporary [3, 7, 18, 23, 31]. Benign MS is an a posteriori diagnosis.

A final point to consider is the fact that the definition for benign MS most commonly used in the literature is based on the EDSS score and therefore mainly the physical disability involved is considered. It is known that cognitive symptoms, depression, fatigue and pain can greatly affect the quality of life of MS patients, but most of these factors are underestimated in the EDSS scale [20, 24, 32, 33].

In our benign MS series, half of the patients evaluated with the MFIS test reported fatigue, which is comparable to the results for benign MS patients reported by other authors [20, 31–34].

Depression is commonly reported in the MS population, with a prevalence of approximately 50% [34]. In Benign MS patients, the percentage varies between 19 and 55% depending on the study and the questionnaire used [20, 32, 33, 35]. In our study, depression was found in 22% of patients with benign MS, which is consistent with the figures obtained in the literature.

Cognition was evaluated in our study via the SDMT. The prevalence of cognitive impairment in benign MS patients varies between 8 and 47% depending on the study [14, 20, 32, 33, 35, 36]. In our study, 83% of the benign MS patients presented with cognitive impairment. In the control group (matched by sex, age and EDSS score but with shorter MS evolution), 55% of the participants had cognitive impairment. The difference did not reach statistical significance. However, differences in the mean SMDT score between the two groups were statistically significant. With a larger number of patients, the differences in the percentage of patients with cognitive impairment may have reached statistical significance.

These results suggest that even when the EDSS score is low in this group, cognitive impairment is notable. Additionally, the longer the duration of MS, the greater the degree of cognitive impairment. Studies with a larger number of patients are needed.

The limitations of our study include the small number of patients for whom the test was performed and that only one evaluation was performed. Study findings suggest a cognitive decline in Benign MS patients over time [37]. Therefore, performing more evaluations throughout the evolution of MS to obtain more accurate results is desirable.

Another limitation is that our study is not a population-based study; therefore, it may not be representative of our real benign MS population. Nonetheless, our results are in line with those of other population-based studies [22], probably because our clinic is the reference centre for demyelinating diseases in a health district and because close monitoring of patients leads to few losses to follow-up.

It should be noted that the patients were 20 years older at the end of the study. Consequently, the age of the patients may have influenced the results, particularly in terms of brain atrophy and volume loss. In the case of cognitive impairment, a control group matched for age and sex has been used to minimize the effect.

In conclusion, the majority of MS patients maintained a benign status even after 30 years of disease evolution. However, cognitive impairment in this group is notable.

## Data Availability

The data that support the findings of this study are available from the corresponding author upon reasonable request.
